# Myocardial oxygenation is impaired in advanced chronic kidney disease and renal transplant patients

**DOI:** 10.1186/1532-429X-17-S1-P116

**Published:** 2015-02-03

**Authors:** Susie F Parnham, Jonathan M Gleadle, Darryl Leong, Suchi Grover, Rebecca Perry, Craig Bradbrook, Richard J Woodman, Carmine De Pasquale, Joseph Selvanayagam

**Affiliations:** Cardiovascular Medicine, Flinders Medical Centre, Bedford Park, SA Australia; Renal Medicine, Flinders Medical Centre, Bedford Park, SA Australia; School of Medicine, Flinders University, Bedford Park, SA Australia; Population Health Research Insititute, Hamilton, ON Canada

## Background

Coronary artery disease (CAD) and left ventricular hypertrophy are prevalent in the chronic kidney disease (CKD) and renal transplant population. Advances in cardiovascular magnetic resonance (CMR) with the blood oxygen level-dependent (BOLD) technique provides unprecedented capability to assess myocardial oxygenation as a measure of ischaemia. We hypothesised that myocardial oxygenation would be reduced in advanced CKD and renal transplant patients and may provide a novel strategy for assessing myocardial ischaemia.

## Methods

We prospectively studied 20 advanced CKD subjects (8 dialysis group with median eGFR 9.5 (range 5-37) ml/min and 12 CKD group with median eGFR 14 (range 8-18) ml/min), 8 renal transplant (RT) recipients with median eGFR 74.5 (range 57-114) ml/min and 7 hypertensive (HT) controls with median eGFR 107 (range 57-144) ml/min. All patients were asymptomatic for CAD and none had prior history of CAD. All groups had cine and BOLD CMR at 3T, and RT and HT groups also had late gadolinium CMR to assess infarction/replacement fibrosis. CKD group additionally underwent 2D echocardiography strain to assess fibrosis. Myocardial oxygenation was measured at rest and under stress with adenosine (140 µg/kg/min) using BOLD Signal Intensity (SI). Analyses were performed using linear mixed models.

## Results

A total of 1074 myocardial segments of the advanced CKD group [522 myocardial segments of dialysis group and 552 myocardial segments of CKD group], 456 myocardial segments of RT and 324 myocardial segments of HT controls were analysed and compared using linear mixed modeling. Mean interventricular septal thickness and left ventricular mass indexed to body surface area was similar between the groups (LV septum advanced CKD 1.2 ± 0.3 cm vs RT 1.2 ± 0.2 cm vs HT 1.1 ± 0.3 cm, p>NS; LV mass index advanced CKD 76 ± 22 g/m2vs RT 67 ± 9 g/m2 vs HT 63 ± 9 g/m2, p>NS). None of the advanced CKD group had impaired global longitudinal strain (GLS) (mean GLS -18.39) and none of the RT/HT groups had late gadolinium hyperenhancement. The mean BOLD SI change was lower in advanced CKD and RT groups compared to HT controls (-0.75 ± 8.82 versus 15.86 ± 9.56, p<0.0001 and 6.57 ± 6.99 versus 15.86 ± 9.56, p=0.033, respectively). The global myocardial BOLD SI change was also lower in the advanced CKD subjects compared to RT recipients (p=0.045). In the advanced CKD and RT groups, the BOLD SI Change was associated with eGFR (β= 0.1, 95%CI= 0.03 to 0.17, p<0.01).

## Conclusions

Our study suggests myocardial oxygenation is impaired in advanced chronic kidney disease patients and renal transplant recipients, and unlikely to be related to LVH or myocardial scarring. The impaired myocardial oxygenation may be associated with declining renal function. Non-contrast BOLD CMR is a promising tool to detect myocardial ischaemia in advanced chronic kidney disease population.

## Funding

None.

**Figure 1:**
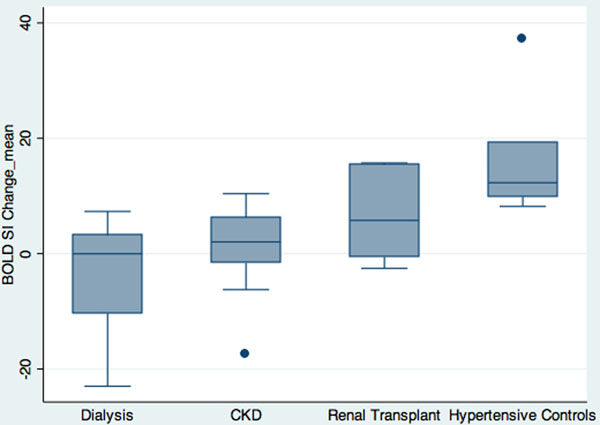
Figure 1

